# Short-Term Outcomes of Digitalis Silicone Implant for Proximal Interphalangeal Joint Osteoarthritis and a Proposed Functional-Radiological Classification

**DOI:** 10.1016/j.jhsg.2025.100911

**Published:** 2026-02-12

**Authors:** Sergi Barrera-Ochoa, Melissa Bonilla-Chaperon, Leobardo Alexis Alvarez-Villalobos, Neri Alejandro Alvarez-Villalobos, Gerardo Mendez-Sanchez

**Affiliations:** ∗ Institut de la Mà, Hospital Universitari General de Catalunya, Barcelona, Spain; † Facultad de Medicina, Universidad Autónoma de Nuevo Leon, Monterrey, Mexico; ‡ Knowledge and Evaluation Research Unit, Mayo Clinic, Rochester, MN

**Keywords:** Degenerative arthritis, Interphalangeal finger joint, Joint prosthesis, Osteoarthritis, Small joint arthritis

## Abstract

**Purpose:**

To evaluate the clinical and functional outcomes of a novel silicone implant (BRM Digitalis) for proximal interphalangeal joint (PIPJ) osteoarthritis and to propose a novel functional-radiological classification system (PIP-Kellgren) adapted from the Kellgren-Lawrence scale.

**Methods:**

This retrospective longitudinal study included 33 patients with symptomatic PIPJ osteoarthritis treated using the BRM Digitalis silicone implant, with a minimum follow-up of 24 months. Subjective outcomes were assessed using the *Quick*DASH (Disabilities of the Arm, Shoulder, and Hand) questionnaire, visual analog scale for pain, and a seven-item Likert satisfaction score. Objective clinical and radiographic data were collected by measuring range of motion (ROM), extension lag, grip and pinch strength, pulp-to-palm distance, and evaluating implant position and integrity on final radiographs.

**Results:**

Statistically significant improvements were observed in total ROM, extension lag, grip strength, and *Quick*DASH and visual analog scale scores. No implant fractures or infections were recorded. The average Likert satisfaction score was 3.97 of 5 (range, 3−5). Using the proposed PIP-Kellgren functional-radiological classification, grade 4 patients showed considerably greater improvements in ROM and *Quick*DASH compared with grade 3, with no differences in satisfaction. The reintervention rate was higher in grade 4.

**Conclusions:**

The BRM Digitalis silicone implant offers consistent improvements in pain, motion, and strength for patients with advanced PIPJ osteoarthritis. The proposed PIP-Kellgren functional-radiological classification system may help stratify surgical candidates and standardize severity assessment.

**Type of study/Level of evidence:**

Therapeutic IV.

Osteoarthritis of the proximal interphalangeal joint (PIPJ OA) is a prevalent degenerative condition that can considerably impair hand function, particularly in patients whose daily or professional activities require precision and dexterity. It is one of the most frequent causes of functional disability in the aging hand, and its treatment remains a clinical challenge. Among various therapeutic options, silicone implants have remained a widely used surgical solution since their introduction by Swanson in 1962.^1^ These implants primarily function as passive spacers through a process of encapsulation, in which a fibrous capsule forms around the prosthesis. This biological response provides stabilization and pain relief while preserving a moderate range of motion (ROM)—without the need for osseointegration.^1^^,^^2^ The absence of osseointegration simplifies implantation and reduces complications associated with bone integration.^1^, ^2^, ^3^

Despite these advantages, conventional silicone implants present biomechanical limitations, particularly in correcting axial misalignment and ensuring rotational stability. These limitations are especially critical in radial digits, where precise alignment is essential for coordinated movement.^4^^,^^5^ Moreover, multiple studies have reported complications such as joint subluxation, dorsal angulation, and progressive deformity over time.^2^^,^^3^ In response, a newly designed silicone implant—the Digitalis implant (BRM Extremities, Italy)—was developed to address these shortcomings.

Although surgical treatment of PIPJ OA is increasingly common, there is no established classification system to objectively assess disease severity or guide treatment selection. In contrast, the Kellgren-Lawrence scale is widely used for staging knee osteoarthritis by integrating radiographic and clinical factors.^6^^,^^7^ Despite multiple series reporting the influence of deformity and subluxation on surgical outcomes, no comprehensive classification has yet been proposed for the PIPJ.^4^^,^^8^ Given the structural and biomechanical similarities between the knee and the PIPJ—both being uniaxial, load-transmitting joints—we developed a novel functional-radiological classification system, termed PIP-Kellgren (Table 1). This five-grade model incorporates pain intensity, joint mobility, angular deformity, and radiographic findings to standardize severity assessment and support clinical decision-making.

**Table 1:** PIP-Kellgren Classification

Functional Grade	Functional Description	Radiographic Osteoarthritis Equivalent (PIP-Kellgren)
Grade 0	No pain or limitation (VAS = 0, ROM > 70°, no deformity)	Grade 0: no radiographic signs
Grade 1	Minimal symptoms (VAS < 3, ROM > 60°, coronal deformity < 5°)	Grade 1: doubtful osteophytes, preserved joint space
Grade 2	Mild/moderate pain (VAS 4–6), ROM > 45°, no subluxation or relevant deformity	Grade 2: defined osteophytes, mild joint space narrowing
Grade 3	Moderate pain (VAS 6–8), ROM 30°–45°, coronal deformity < 15°, no subluxation	Grade 3: moderate joint space narrowing, mild subchondral sclerosis
Grade 4	Severe pain (VAS > 8), ROM < 30°, subluxation or coronal angle ≥ 15°	Grade 4: severe joint space narrowing, deformity or collapse

PIP-Kellgren functional-radiological classification system for proximal interphalangeal joint osteoarthritis. Coronal deformity: angular deviation on anteroposterior radiograph.

ROM, range of motion (in degrees); VAS, visual analog scale (0 = no pain, 10 = worst imaginable pain).

This study aimed to present the short-term clinical and radiographic outcomes, with a minimum 2-year follow-up, in a cohort of patients with PIPJ OA treated using this newly designed silicone implant. Additionally, we introduce and explore the application of a functional-radiological classification system (PIP-Kellgren), evaluating its correlation with clinical outcomes and its potential usefulness in guiding treatment strategies.

## Materials and Methods

### Study design and patients

This ambispective cohort study included 33 adult patients diagnosed with symptomatic PIPJ OA unresponsive to conservative management, who underwent silicone arthroplasty using the BRM Digitalis implant. Surgical procedures were performed between January 2018 and December 2022 by two experienced hand surgeons at our institution, and data collection concluded in February 2025.

Inclusion criteria were as follows: (1) age ≥18 years, (2) at least 24 months of postoperative follow-up, and (3) complete clinical and radiographic records. Patients were excluded if they had concomitant procedures, or contralateral PIPJ OA exceeding Kellgren-Lawrence stage II.

The study protocol was approved by the Institutional Review Board, and written informed consent was obtained from all participants. Ethical approval for this study was obtained from Hospital Universitari General de Catalunya’s ethics committee.

Collected variables included demographic data (age, sex, hand dominance, affected digit, and surgical approach), operative data (implant size, operative time, intraoperative complications), and clinical outcomes: pre- and postoperative ROM, extension lag, grip strength, key and tip pinch strength, pulp-to-palm distance, pain assessment via 10-point visual analog scale (VAS) at rest and during effort, and functionality using the *Quick*DASH score. Radiological evaluations assessed implant position and integrity. Coronal alignment was quantified on anteroposterior (AP) radiographs. Sagittal alignment was assessed qualitatively on lateral radiographs for evidence of volar/dorsal translation or loss of joint congruity. Patient satisfaction was recorded on a 0−10 scale.

### Surgical technique

All surgical procedures were performed under regional anesthesia using either a dorsal or volar approach, depending on patient anatomy and surgeon preference.

#### Dorsal approach

A gently curved dorsal incision was made directly over the PIPJ. Dissection continued until the extensor tendon complex was reached. A dorsal extensor-splitting technique was employed, elevating the extensor tendon and central slip to expose the joint. Depending on the degree of preoperative contracture, collateral management was performed on the tight side of the deformity. To improve exposure, we routinely released the accessory collateral ligament. In cases with greater deformity, we added a partial release of the proper collateral ligament at its distal bony insertion on the base of the middle phalanx (P2)—never complete—to facilitate coronal balancing.

#### Volar approach

**Figure 1:**
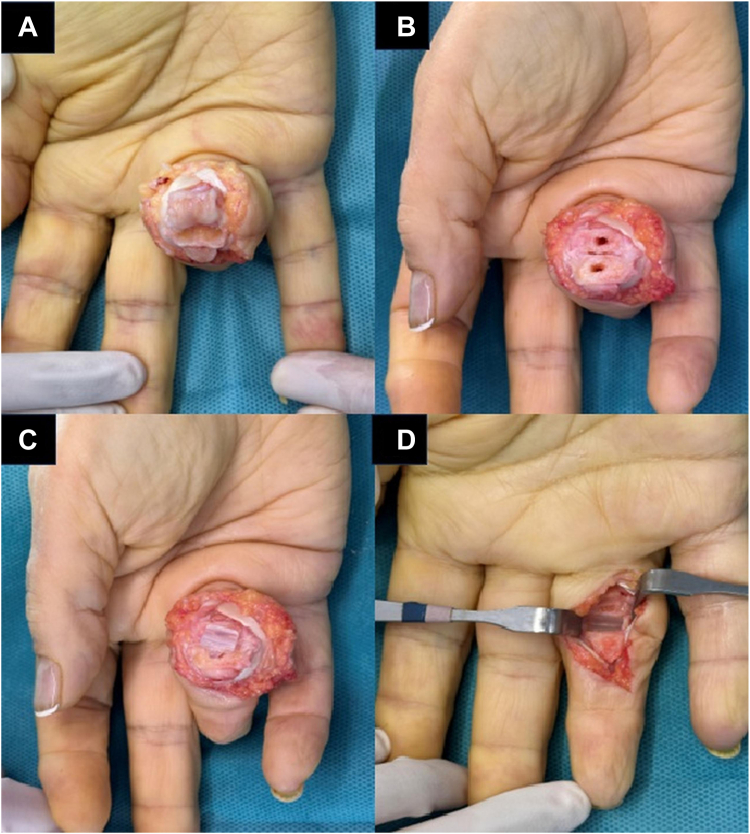
Volar approach for proximal interphalangeal joint (PIPJ) arthroplasty. **A** Arthritic joint exposure through volar approach, after A2-A3-A4 flap and the deinsertion of the volar plate. **B** Bone tunnel placement. **C** Implant positioning. **D** Final reduction.

For the volar approach (Fig. 1), a Bruner-type incision was made over the volar aspect of the PIPJ. After the skin incision, the A2, A3, and A4 pulleys were incised and reflected, and the flexor tendons were retracted laterally to gain access to the joint. The volar plate was incised and reflected distally (Fig. 1A). In cases with notable contracture, we performed a controlled shotgun maneuver. Exposure was obtained through a distal collateral release: routine distal release of the accessory collateral (from the volar plate/P2) and, in greater deformity, a limited distal release of the proper collateral at its bony insertion on P2.

#### Osteotomy of the joint surfaces

With soft tissue protection in place, an oscillating saw was used to resect the condylar head of the proximal phalanx. The base of the middle phalanx was contoured to correct surface irregularities caused by arthritic changes. Any osteophytes or bony spurs from the joint surfaces, as well as from the flexor and extensor mechanisms, were carefully removed.

#### Preparation of the medullary canals

**Figure 2:**
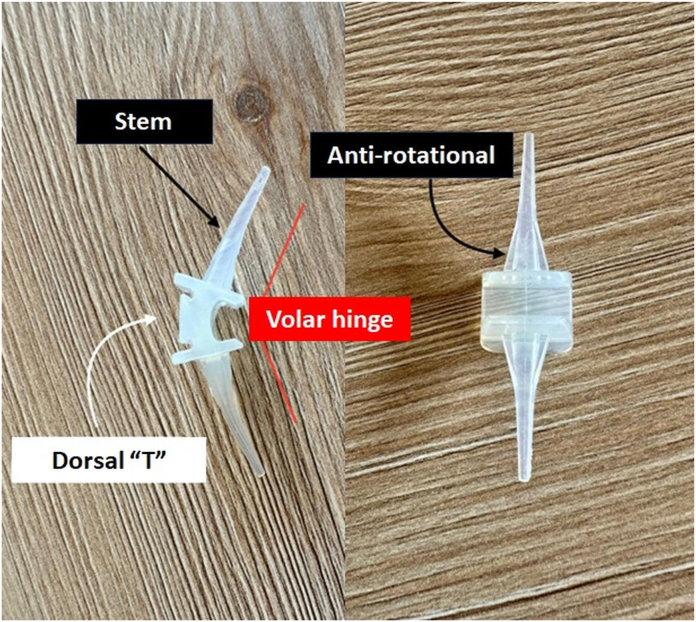
Implant design. Digitalis (BRM Extremities, Italy) design: volar hinge allowing up to 90° flexion (red lines), “dorsal T” for hyperextension prevention (white arrow); pseudoconical, antirotational stems (black arrow).

The medullary canals of both the proximal and middle phalanges were manually located using a surgical awl. Once identified, the reamer was mounted on the handle and introduced into the canal to the depth corresponding to the selected implant size— marked on the instrument (Fig. 2B). A rasp was then used to complete the canal preparation, keeping the rasp edges parallel to the corresponding bony borders. The rasp was advanced until the preselected depth was reached. Adjustments to the tightness of the reamer or rasp were made using the key on the multisize trial PIP instrument (primarily designed for dorsal approach arthroplasty), as needed.

#### Trial and implant fitting

The trial spacer corresponding to the selected implant size was inserted into the prepared joint. The surgeon assessed its conformity to the resected surfaces, checking for mobility, alignment, and stability. The finger was flexed and extended to confirm implant retention throughout the ROM. Tissue balancing was adjusted as needed to avoid overstuffing and to improve coronal alignment when indicated. In general, the lax side was slightly retensioned with an intraligamentous 4-0 Monocryl stitch. Only occasionally, on the tight side (the direction of the preoperative deviation), a portion of the accessory collateral was reanchored to its volar plate insertion and gently retensioned to aid alignment.

#### Final implant insertion and closure

For dorsal cases, after verifying trial fitting, the final implant was inserted. The extensor mechanism was closed with 4-0 absorbable sutures, and the skin was approximated using 5-0 nonabsorbable sutures. A soft dressing was applied, maintaining the PIPJ in slight flexion (5° to 10°).

For volar cases, after implant insertion (Fig. 1C, D), the volar plate was reattached with absorbable sutures to fully cover the implant. The reflected A2, A3, and A4 pulleys were reapproximated with 4-0 absorbable sutures to restore tendon continuity. The skin was closed with 5-0 nonabsorbable sutures, and a dressing was applied while positioning the joint in a relaxed state. A dorsal splint was applied, maintaining the PIPJ in slight flexion (5° to 10°).

### Postoperative care

Patients were reviewed at 1 week, 2 weeks, 6 weeks, 3 months, 6 months, 12 months, and annually thereafter. During the first postoperative week, patients performed active flexion and extension while hyperextension was limited with a dorsal splint. At 1 week, a lighter dressing was applied to facilitate increased ROM. At 2 weeks, sutures were removed and rehabilitation began with a focus on edema control and the progressive restoration of full joint mobility. A dynamic night-splinting program was initiated using elastic-tension digital neoprene orthoses to promote extension. Grip-strengthening exercises were introduced at approximately 4−6 weeks, once near-maximal ROM had been achieved.

### Implant design and biomechanical features

The implant used in this study was the Digitalis PIP silicone implant, a one-piece spacer made of medical-grade silicone elastomer, specifically designed for total arthroplasty of the PIPJ. The implant is available in four ambidextrous sizes—small, medium, large, and extra-large.

The implant features specific biomechanical and geometric characteristics, as illustrated in Fig. 2.

1. A central hinge allows controlled flexion up to 90°.
2. A dorsal T-shaped geometry functions as a mechanical stop to prevent hyperextension while enhancing lateral and medial stability.
3. Symmetrical, pseudoconical stems taper distally, preserving the medullary canal and reducing the risk of rotational micromotion.
4. The stems incorporate three longitudinal grooves—two dorsal and one palmar—which improve fixation through increased frictional contact while allowing limited axial gliding. This design aims to avoid the so-called piston effect, which has been associated with implant fatigue and stress transmission in conventional silicone arthroplasties.
5. The implant is noncemented and nonpress-fit, relying on balanced mechanical engagement and soft tissue encapsulation for stabilization.

Mechanical fatigue testing under simulated worst-case scenarios demonstrated good hinge durability, withstanding >10 million flexion-extension cycles without evidence of structural failure, hinge fracture, or silicone degradation.^9^

In this study, each of these design targets was operationalized using coronal angulation, extension lag, ROM, patient-reported outcomes, reintervention, and radiographic integrity.

### Clinical and radiographic assessments

Functional and clinical outcomes were evaluated both before surgery and at final follow-up. Objective assessments included PIPJ ROM—measured with a goniometer (extension and total arc)—as well as extension lag (in degrees), and grip and pinch strength (in kilograms), quantified using a dynamometer (Baseline® Evaluation Instruments, White Plains, NY). Pulp-to-palm distance was measured in centimeters.^10^

Functional disability was assessed using the *Quick*DASH questionnaire, with scores ranging from 0 (no disability) to 100 (maximum disability).^11^ The Michigan Hand Outcomes Questionnaire was performed on all patients, consisting of 37 items assessing six domains on both hands: overall hand function, activities of daily living, work, pain, aesthetics and satisfaction.^12^ Pain intensity was quantified using a 10-point VAS, separately at rest and during effort (0 = no pain; 10 = worst imaginable pain).^13^ When applicable, outcomes were compared with the contralateral, nonoperated hand. Postoperative evaluations were conducted by an independent senior hand surgeon not involved in the original procedures.

### Functional-radiological classification of PIPJ OA (PIP-Kellgren)

To stratify disease severity, our proposed functional-radiological classification system, termed PIP-Kellgren, was applied retrospectively (Table 1). This model was adapted from the Kellgren-Lawrence classification used in knee osteoarthritis and integrates clinical and imaging criteria. Patients were assigned to one of five grades (0−4) based on the following parameters:

1. Pain intensity, measured by VAS during effort.
2. Preoperative ROM, measured with a goniometer.
3. Presence of coronal angular deformity, identified on AP radiographs.
4. Presence of joint subluxation, assessed qualitatively on lateral radiographs.

This classification was used to explore potential correlations between baseline severity and clinical outcomes.

### Radiographic evaluation

**Figure 3:**
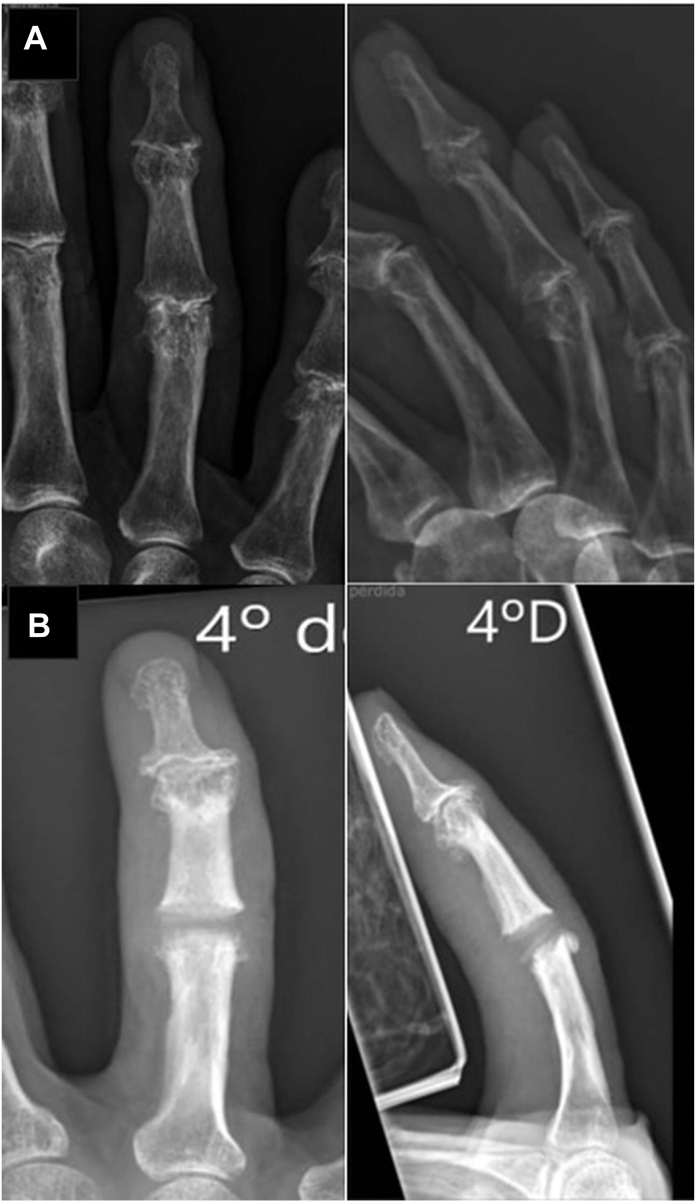
Radiographic assessment. A 74-year-old woman’s before surgery **A** and 30-month postoperative images **B** after volar approach proximal interphalangeal joint (PIPJ) arthroplasty.

Standardized AP and lateral radiographs of the operated hand were obtained before surgery and at final follow-up (Fig. 3A, B). The following radiographic parameters were assessed:

Coronal angulation of the PIPJ (in °), measured as the angle between the longitudinal axes of the proximal and middle phalanges. Sagittal alignment was assessed qualitatively (yes/no) on lateral radiographs for evidence of volar/dorsal translation or loss of joint congruity.

Signs of implant-related complications, including loosening, subsidence, fracture, and/or periprosthetic bone erosion. Implant integrity and fixation were evaluated on standardized AP and lateral radiographs at each follow-up and compared with the immediate postoperative images. Fracture was defined as hinge discontinuity or new deformation of the spacer contour. Loosening/failure surrogates included progressive periprosthetic radiolucency >1 mm at the stem–bone interface on sequential films, endosteal scalloping/periprosthetic erosion, axial migration/subsidence (change in the spacer position relative to fixed P1/P2 landmarks), and recurrent malalignment after initial correction. Additional views were obtained when needed; cross-sectional imaging was used selectively if plain films were inconclusive.

### Patient-reported outcomes

Subjective satisfaction was measured using a seven-item Likert-type questionnaire,^3^ covering the following domains: overall satisfaction, hand function, willingness to undergo surgery again, pain, joint mobility, aesthetic appearance, and likelihood of recommending the procedure to others. Each item was scored on a five-point scale (1 = strongly disagree; 5 = strongly agree), and a global satisfaction score was calculated as the mean across all items.

### Definition of implant survival and reintervention

Implant survival was defined as the absence of revision surgery requiring removal of the prosthesis. Reoperations not involving implant exchange (eg, extensor tenolysis) were recorded separately and were not considered implant failures. The total number of reinterventions was documented and analyzed descriptively.

### Statistical analysis

Statistical analysis was performed using Python 3.11 with the SciPy library. Continuous variables were expressed as mean ± standard deviation. Paired *t* tests were applied to compare preoperative and postoperative values, and, when applicable, postoperative outcomes were compared with measurements from the contralateral hand. Subgroup analyses included stratification by treated digit (index-2, middle-3, ring-4, or small-5) for ROM and *Quick*DASH scores. The proportion of patients achieving predefined clinical thresholds was also analyzed: ROM ≥ 70°, *Quick*DASH ≤ 25, and VAS during effort ≤ 1. Additionally, the correlation between postoperative coronal angulation and global satisfaction score (Likert scale) was assessed using Wilcoxon’s correlation coefficient. A *P* < .05 was considered statistically significant.

## Results

### Demographics and surgical characteristics

Demographic and general features are presented in Table 2.

**Table 2:** General Features

N = 33	Value
Age (average)	62.72 ± 8.45
Follow-up period (average, in mo)	27.27 ± 1.97
Duration of operation (average, min)	51.39 ± 2.93
Sex	
Female (%)	24 (72.7%)
Male (%)	9 (27.3%)
Type of occupation	
Manual work (%)	9 (27.3%)
Nonmanual work (%)	12 (35.4%)
Retired (%)	13 (35.4%)
Operated side (dominant vs nondominant)	
Dominant (%)	28 (84.85%)
Nondominant (%)	5 (15.15%)
Operated finger	
2 (%)	1 (3.03%)
3 (%)	19 (57.57%)
4 (%)	9 (27.27%)
5 (%)	4 (12.12%)
Type of approach	
Volar (%)	31 (94.9%)
Dorsal (%)	2 (6.1%)
Etiology	
Posttraumatic (%)	1 (3.0%)
Degenerative (%)	29 (87.9%)
Rheumatoid (%)	3 (9.1%)

The implant demonstrated high short-term durability. Only one documented implant failure required conversion to PIP arthrodesis at 32 months for persistent pain and stiffness, without radiographic evidence of implant fracture, migration/subsidence, or progressive periprosthetic radiolucency. One additional patient underwent extensor tenolysis at 24 months for postoperative adhesions after a dorsal approach; this reoperation did not involve implant exchange or removal. Accordingly, the overall implant survivorship (freedom from implant revision) was 97% at 2 years, and the reintervention rate was 6.1%.

### Functional and clinical outcomes

Postoperative improvements were statistically and clinically significant across all functional domains. A high proportion of patients met clinically relevant thresholds for arc of motion (≥70°), minimal disability (*Quick*DASH ≤ 25), and low pain during effort (VAS ≤ 1) (Table 3).

**Table 3:** Score Comparisons

Scores	Before Surgery n = 33	After Surgery n = 33	*P*	CI (95%) for Δ	Cohen's d
*Quick*DASH	73.64 ± 5.63	23.79 ± 5.59	<.001	(49.31−50.39)	32.7
VAS rest	6.36 ± 0.74	0.61 ± 0.61	<.001	(5.48−6.04)	7.27
VAS effort	7.85 ± 0.51	0.76 ± 0.61	<.001	(6.83−7.35)	9.81
MHOQ∗	40.91 ± 4.71	80.91 ± 4.71	<.001	-	Inf

CI, 95% confidence interval; *Quick*DASH, Quick Disabilities of the Arm, Shoulder, and Hand (0 = no disability, 100 = maximum disability); MHOQ, Michigan Hand Outcomes Questionnaire (0 = poor function, 100 = excellent function); VAS, visual analog scale (0 = no pain, 10 = worst imaginable pain); Δ, change (after surgery – before surgery).

∗ MHOQ was analyzed using the Wilcoxon signed-rank test because of nonparametric distribution. All other comparisons were performed using paired *t* tests. Variable was analyzed under a nonparametric test (Wilcoxon's).

### Satisfaction and radiographic findings

Radiographic assessment demonstrated notable correction of coronal angulation, with no evidence of implant fracture, loosening, or periprosthetic bone erosion. A moderate, nonsignificant negative correlation was found between final coronal angulation and overall satisfaction scores. In grade 4 cases (n = 12), the joint was reduced intraoperatively. Postoperative lateral radiographs showed no persistent or recurrent sagittal subluxation, and no clinical instability was recorded at final follow-up (Table 4).

**Table 4:** Goniometry/Dynamometry Measurements

Measurement	Before Surgery n = 33	After Surgery n = 33	*P*	CI (95%) for Δ	Cohen's d
Coronal angulation	12.73 ± 3.87	7.36 ± 3.22	<.001	(4.21−6.52)	1.65
Δ - Coronal angulation	5.36 ± 3.26				
PIP ROM∗	39.70 ± 5.14	74.70 ± 5.14	<.001	-	inf
Extension lag	17.56 ± 4.86	8.03 ± 4.50	<.001	(10.66−8.43)	3.03
Total ROM	141.51 ± 9.26	191.82 ± 9.42	<.001	(48.98−51.63)	13.46
Pulp-to-palm distance (PPD) (cm)	3.30 ± 0.68	0.61 ± 0.61	<.001	(2.45−2.94)	3.94
Grip (kg)	22.76 ± 3.64	29.27 ± 3.76	<.001	(5.89−7.14)	3.72

Coronal angulation: angle between the longitudinal axes of the proximal and middle phalanges; extension lag: incomplete active extension of the PIP joint; grip: maximum grip strength in kilograms.

CI, 95% confidence interval; PIP ROM, proximal interphalangeal joint range of motion (flexion-extension arc); PPD: pulp-to-palm distance in centimeters; Δ, change (after surgery – before surgery).

∗ PIP ROM was analyzed using the Wilcoxon signed-rank test because of nonparametric distribution. All other comparisons were performed using paired *t* tests.

Patient-reported satisfaction was high. The average score on the global Likert scale was 3.97 ± 0.39 of 5, with most patients reporting that they were satisfied with the outcome, would undergo the procedure again, and would recommend it to others. Greater variability was noted in responses related to aesthetics and joint motion.

### Stratification by disease severity (PIP-Kellgren classification)

Patients were retrospectively categorized using the PIP-Kellgren classification. All were classified as grade 3 (63.6%) or grade 4 (36.4%), reflecting the advanced nature of disease in this surgical cohort.

Patients in grade 4 exhibited considerably greater improvements in ROM and *Quick*DASH scores compared to those in grade 3, as well as a higher reintervention rate (Table 5).

**Table 5:** Clinical and Functional Outcomes by PIP-Kellgren Grade

Outcome	Grade 3 (n = 21)	Grade 4 (n = 12)	*P*	95% CI for Δ (Grade 4–Grade 3)
ΔROM (°)	33.8 ± 2.7	36.6 ± 2.4	.0001	(1.04–4.56)
Δ*Quick*DASH	47.0 ± 4.1	53.3 ± 4.7	.01	(1.30–11.30)
Satisfaction (mean)	3.96 ± 0.39	3.97 ± 0.41	.95	(−0.30 to 0.32)
Reintervention rate (%)	0	16.70	—	—

Comparative outcomes between PIP-Kellgren grade 3 and grade 4 patients; ΔROM, change in range of motion (in degrees); Δ*Quick*DASH, change in Quick Disabilities of the Arm, Shoulder, and Hand score (0 = no disability, 100 = maximum disability); Likert Satisfaction: average score on a seven-item Likert scale (1 = strongly disagree, 5 = strongly agree); reintervention: proportion of patients requiring additional surgery.

## Discussion

This study demonstrates that silicone implant using the BRM Digitalis implant leads to notable clinical improvements in patients with PIPJ OA. Participants experienced substantial pain relief, meaningful gains in grip strength and ROM, and high satisfaction levels. The observed 49.85-point reduction in *Quick*DASH scores and the 35° increase in arc of motion exceed established thresholds for clinical relevance, supporting the utility of this implant in the management of advanced PIPJ degeneration.

Our results compare favorably with previous series using Swanson and NeuFlex implants, which typically reported ROM gains between 30° and 34°, and more modest changes in *Quick*DASH scores.^1^^,^^2^^,^^14^ Moreover, the complication rate in our cohort (6.1%) was lower than the 14% reported in the literature.^8^ Importantly, there were no cases of implant fracture or radiographic loosening at 2 years, and the overall implant survival rate reached 96.97%, surpassing the 84% to 90% survival rates described in longer-term studies.^3^

The design characteristics of the Digitalis implant likely contribute to these favorable outcomes. Features such as the central hinge, symmetrical stems, and dorsal T-stop improve resistance to axial rotation and hyperextension. Additionally, controlled stem gliding reduces the piston effect and may lessen hinge fatigue over time. These biomechanical advantages are particularly relevant in radial digits, where precise alignment is critical.^4^^,^^15^ The notable postoperative correction of coronal deviation supports the hypothesis that implant geometry contributes to restoring joint alignment.

Our findings also reinforce the growing consensus favoring the volar surgical approach. In our series, volar access was employed in nearly all cases, with no subsequent need for tenolysis. Extension was preserved or improved with no deficits attributed to the extensor mechanism, even though a natural, slight flexion position was held during the first two weeks. In contrast, one of the two dorsal approach cases required extensor release. This aligns with previous studies,^2^^,^^16^, ^17^, ^18^, ^19^, ^20^, ^21^ which highlight the benefits of preserving the extensor mechanism to optimize rehabilitation and reduce complications. In our study, because of the small number of dorsal cases, no formal statistical comparison was performed between the two approaches.

A novel contribution of this study is the introduction and application of the PIP-Kellgren classification—a five-grade system integrating functional and radiological criteria. Although patient satisfaction did not differ considerably between grades 3 and 4, those classified as grade 4 demonstrated greater functional improvements, likely reflecting more severe baseline impairment. The higher reintervention rate observed in this group underscores the complexity of advanced disease and supports the classification’s potential value for preoperative planning and patient counseling.

To our knowledge, this is the first structured system specifically developed to stage PIPJ OA. While earlier studies have acknowledged the prognostic relevance of deformity and subluxation,^4^^,^^8^ no formal framework had been established to quantify disease severity and correlate it with outcomes. The proposed PIP-Kellgren classification may help fill this gap; however, further validation of its external applicability is needed.

Interestingly, no association was found between implant size and the specific digit treated, reinforcing the notion that implant selection should be guided intraoperatively by canal morphology rather than finger identity. This observation is consistent with previous findings and highlights the importance of individualized surgical planning in silicone arthroplasty.^5^

Although this study is classified as Level IV evidence, its methodological rigor—including prospective follow-up, standardized assessments, and independent outcome evaluations—enhances the reliability and clinical relevance of the findings. In implant research, where randomized trials are often limited by ethical or logistical barriers, well-conducted cohort studies continue to provide valuable insights for clinical practice.

Furthermore, the mean time to return to work (12.7 weeks) aligns with contemporary implant cohorts and supports the procedure’s capacity to restore functional autonomy efficiently. Although rarely reported, this parameter may gain increasing relevance in outcome evaluations for working-age populations.

This study has several limitations. Its retrospective design and sample size limit the generalizability of the results. Given the modest sample size, this study is not powered to demonstrate superiority versus other implants. The short-term follow-up (minimum 24 months) does not permit conclusions regarding long-term implant durability. Although coronal correction was quantified, sagittal translation was assessed qualitatively. Future analyses will include a quantitative sagittal metric (eg, percent volar/dorsal translation) to better characterize reduction maintenance. Additionally, the PIP-Kellgren classification was developed retrospectively and has not yet undergone interobserver reliability testing. Last, the small number of dorsal approach cases precluded meaningful comparative analysis between surgical techniques.

In conclusion, BRM Digitalis silicone implant produced robust improvements in pain relief, functional performance, and joint alignment, with high satisfaction and low complication rates in patients with severe PIPJ OA. The implant’s design appears to offer distinct biomechanical advantages, particularly in restoring coronal alignment in anatomically challenging cases. The proposed PIP-Kellgren classification may serve as a useful framework for assessing disease severity and guiding surgical decision-making, though prospective validation in larger cohorts is warranted.

Future studies should focus on validating the PIP-Kellgren scale across multicenter populations prospectively rather than in an exploratory manner, assessing its interobserver consistency, and comparing the performance of the Digitalis implant with other designs over longer follow-up periods. Incorporating automated radiographic analysis tools may further enhance reproducibility and support the development of decision-support models in hand arthroplasty.

## Conflicts of Interest

No benefits in any form have been received or will be received related directly to this article.
